# Current status and prospects of diagnosis and treatment for esophageal cancer with supraclavicular lymph node metastasis

**DOI:** 10.3389/fonc.2024.1431507

**Published:** 2024-10-11

**Authors:** Qingxin Cai, Yingji Hong, Xuehan Huang, Tong Chen, Chuangzhen Chen

**Affiliations:** ^1^ Department of Radiation Oncology, Cancer Hospital of Shantou University Medical College, Shantou, China; ^2^ Shantou University Medical College, Shantou, China

**Keywords:** esophageal cancer, supraclavicular lymph node, American joint committee on cancer (AJCC) 8th staging systems, Japan esophageal society (JES) 12th edition classification, chemoradiotherapy, surgery

## Abstract

Patients with supraclavicular lymph node (SLN) metastasis from esophageal cancer encounter significant variations in treatment approaches due to differences in pathological subtypes and the lack of a unified regional staging system between East Asian and Western countries. The Tiger study aims to develop an internationally recognized staging system and to delineate the extent of regional lymph node dissection. In the context of esophageal squamous cell carcinoma (SCC) with SLN metastasis, the treatment paradigms from East Asia offer valuable insights. The Japan Esophageal Society (JES) 12th edition staging system guides a tailored comprehensive treatment strategy, emphasizing either radiotherapy and chemotherapy or surgical intervention. In contrast, esophageal adenocarcinoma (AC) predominates in Western countries, where the 8th edition of the American Joint Committee on Cancer (AJCC) staging system classifies SLN metastasis as a distant metastasis, advocating for systemic therapy as the primary treatment modality. Nonetheless, compelling evidence suggests that a multidisciplinary treatment approach, incorporating either radiotherapy and chemotherapy or surgery as the initial treatment, can yield superior outcomes for these patients compared to chemotherapy alone.

## 1 Introduction

In 2020, esophageal cancer was the seventh most commonly diagnosed cancer globally, and the sixth leading cause of cancer-related mortality (1). Post-surgical supraclavicular lymph node (SLN) metastasis occurs in 14.5-25% of patients with esophageal cancer (2–4). Currently, there is no unified clinical staging for esophageal cancer patients with SLN as the solitary metastasis site. The 8th edition of the American Joint Committee on Cancer (AJCC) staging system categorizes SLN metastasis as M1, equating to stage IVB (5). The National Comprehensive Cancer Network (NCCN) guidelines prioritize systemic therapy for all M1 patients. In contrast, the 12th edition of the Japan Esophageal Society (JES) staging system classifies these patients as M1a, with stages ranging from III to IVA (6). The recommended treatment approach in this system involves surgery coupled with perioperative treatment and definitive chemoradiotherapy (dCRT), emphasizing curative intent and a focus on local treatment. The Chinese Society of Clinical Oncology (CSCO) guidelines, which adopt the AJCC staging, recommend similar treatment principles to the NCCN for different stages. This leads to significant discrepancies in staging and markedly different treatment strategies for patients with esophageal cancer and SLN metastasis when different staging systems are applied. This review will compare and discuss the classification of SLNs, staging, treatment strategies, and recent advancements in the management of this patient cohort.

## 2 Attribution of supraclavicular lymph nodes

Anatomically, SLNs are part of the cervical lymph node region. However, due to frequent involvement in thoracic tumors, SLNs are often included in the definition of regional lymph nodes in thoracic tumors. There are variations in the anatomical boundaries of SLNs as defined by different staging systems, as well as differences in the regional lymph node definitions for esophageal cancer (**Table 1**).

**Table 1:** Anatomic boundaries of supraclavicular lymph node.

	AJCC8th-head&neck Vb	2013-IVb (13)	2013-Vc (13)	Michigan -IASLC (18)	JES-104 (20)	Tiger (23)
Cranial	Horizontal plane defined by the lower border of the cricoid cartilage	Caudal border of level IVa (2 cm cranial to sternal manubrium)	Plane just below transverse cervical vessels	Lower margin of cricoid cartilage	Lymph nodes located in the supraclavicular fossa, extending from the lower border of the cricoid cartilage superiorly, to the clavicle inferiorly, including the lower internal deep cervical lymph nodes. The medial boundary is the medial border of the carotid sheath.	
Caudal	Clavicle	Cranial edge of sternal manubrium	2 cm cranial to sternal manubrium, i.e. caudal border of level IVa	Laterally: both clavicles Medially: upper border of manubrium		
Anterior	Posterior border of the sternocleidomastoid muscle	Deep surface of sternocleidomastoid m./deep aspect of clavicle	Skin	–		
Posterior	Anterior border of the trapezius muscle	Anterior edge of scalenius mm. (cranially)/apex of lung, the brachiocephalic vein, the brachiocephalic trunk (right side) and the common carotid artery and subclavian artery on the left side (caudally)	Anterior border of trapezius m. (cranially)/± 1 cm anterior to serratus anterior m. (caudally)	–		
Lateral	–	Lateral edge of scalenius m.	Trapezius m (cranially)/clavicle (caudally)	–		
Medial	–	Lateral border of level VI (pre-tracheal component)/medial edge of common carotid artery	Scalenius m./lateral edge of sternocleidomastoid m, lateral edge of	midline of trachea		

### 2.1 Supraclavicular lymph nodes in head and neck tumors

The cervical lymph node region was initially defined by anatomist Rouvière in 1938 (7). Rouvière believed that SLNs belonged to the deep lymph nodes of the lateral and lower neck, located along the transverse cervical vessels. The American Academy of Otolaryngology-Head and Neck Surgery (AAO-HNS) proposed a division of cervical lymph nodes into 6 levels according to the level-based approach in 1991 (8), it was revised twice in 1998 and 2002 (9, 10), leading to the modified Robbins’ classification. The 7th edition of the AJCC staging in 2010 designated SLNs as a subset of the level Vb region. The 8th edition maintained the cervical lymph node region divisions.

With the enhanced understanding of anatomy, especially lymphatic drainage, and the advancement of imaging techniques, some researchers believe that the anatomical accuracy provided by CT and MRI can solve these problems and improve the accuracy of classification and assessment of metastatic lymph nodes. In 2000, Som first proposed an imaging-based definition of cervical lymph node regions (11), defining SLNs as lymph nodes located at or below the level of the clavicle, lateral to the carotid artery, distinct from regions I-VII. In 2003 (12), major tumor cooperative organizations in Europe and the United States jointly published an imaging-based division of cervical lymph node regions, which was updated in 2013 (13), defining SLNs as the region from the upper edge of the sternum to 2cm above it, divided into IVb (medial supraclavicular group) and Vc (lateral supraclavicular group).

### 2.2 Supraclavicular lymph nodes in thoracic tumors

In the context of thoracic tumors, the classification of SLNs has evolved over time. Initially, Naruke’s 1967 lymph node mapping for lung cancer did not include SLNs (14). The American Thoracic Society (ATS) later incorporated SLNs as Group 1 in their 1983 and 1988 lymph node maps (15, 16), which were not initially recognized in the AJCC’s 2nd edition. The International Association for the Study of Lung Cancer (IASLC) established a more detailed lymph node map in 2009 (17), providing detailed anatomical definitions and becoming the standard for regional lymph node classification in lung cancer. The supraclavicular region is designated as Group 1, including the lower neck, supraclavicular, and sternal lymph nodes.

As radiotherapy techniques like 3D-CRT and IMRT advanced, the University of Michigan provided a CT-based lymph node classification in 2013 to improve target delineation in lung cancer (18).

For esophageal cancer, the JES and the AJCC have different staging systems. JES’s 12th edition considers Group 104 as M1a, a non-regional lymph node, distinguishing it from other distant metastases (M1b). Prior to the 8th edition of the AJCC staging, SLNs were considered regional nodes but were reclassified out of this category in the latest edition (19).

Researchers use CT-based classifications for esophageal cancer lymph nodes (20), often relying on existing cervical or lung cancer lymph node classifications, as specific guidelines for SLNs in esophageal cancer are not fully established.

### 2.3 Current situation

Globally, there’s a noticeable lack of consensus regarding the involvement of SLNs in esophageal cancer research, with interpretations varying and sometimes conflicting. A primary issue is the infrequency of a precise definition for SLN metastasis within the field. The absence of a universally accepted definition often results in vague or unspecified criteria in studies, highlighting an urgent need for standardized terminology. Furthermore, even when using the same staging system, researchers interpret SLN involvement differently. Although the widely used 8th edition of the AJCC staging system for esophageal cancer has improved over the 7th edition by clearly defining lymph node stations specific to esophageal cancer and differentiating them from those in lung cancer, it still does not provide clear anatomical boundaries for lymph nodes in the esophageal cancer region. This has led to some misunderstandings. For example, Xu et al. from China mentioned in their article that SLN metastasis is considered a regional lymph node in the 8th edition of the AJCC staging system (21), and they believe that group 1 of the regional lymph nodes in the 8th edition represents the SLNs. The other two examples are retrospective studies conducted in the Netherlands and Japan, respectively (3, 22), which stated that: In the 7th edition of the AJCC staging system, SLNs are not considered regional lymph nodes in esophageal cancer.

Due to the lack of a unified standard for defining regional lymph nodes in esophageal cancer and the absence of clear anatomical boundaries for lymph nodes, researchers have not reached a consensus on the definition of SLNs and whether they should be classified as regional lymph nodes. The confusion in definitions has resulted in confusing research, which cannot yield clinically significant results. To tackle this issue at its core, the Tiger study was undertaken collaboratively by 50 institutions across multiple countries (23). The study aims to enroll 5000 patients who undergo surgery for resectable esophageal cancer or gastroesophageal junction cancer, with the goal of determining the distribution of lymph node metastasis and developing a globally standardized staging system. This will lay the foundation for establishing the optimal surgical treatment strategy for esophageal cancer patients. The definition of SLNs in this study is based on the 11th edition of the JES staging, specifically JES-104 group: lymph nodes located in the supraclavicular fossa, ranging from the upper border of the clavicle to the lower border of the cricoid cartilage, with the inner border being the inner edge of the carotid sheath.

In light of these efforts, the Tiger study’s partition scheme, endorsed by 18 countries and 50 institutions, is seen as the most rational approach to defining SLNs in esophageal cancer to date. It’s hoped that the study’s outcomes will pave the way for a unified definition of SLN involvement, enabling more consistent data comparison and guiding the development of more effective treatment strategies. Until then, clarity in defining SLNs in research papers is strongly advised.

## 3 Staging and treatment recommendations

### 3.1 Current status

There are two widely used staging systems for esophageal cancer currently, the AJCC 8th edition staging and the JES 12th edition staging (**Table 2**). Patients with SLN metastasis are classified as stage IVB in the AJCC 8th edition staging, but in the JES 12th edition staging, SLN as the solitary distant metastasis are classified as M1a, and the stage can be III-IVA.

**Table 2:** The difference between the JES12th staging and the AJCC8th staging.

JES12th		AJCC8th
	Primary tumor cannot be assessed	Tx
T0	No evidence of primary tumor	T0
T1	Tumor invades the lamina propria, muscularis mucosae, or Submucosa	T1
T1a	Tumor invades mucosa	T1a
T1a-EP	Carcinoma *in situ* (Tis)	Tis
T1a-LPM	Tumor invades lamina propria mucosae (LPM)	–
T1a-MM	Tumor invades muscularis mucosae (MM)	–
T1b	Tumor invades the submucosa (SM)	T1b
T1b-SM1	Tumor invades the upper third of the submucosal layer	–
T1b-SM2	Tumor invades the middle third of the submucosal layer	–
T1b-SM3	Tumor invades the lower third of the submucosal layer	–
T2	Tumor invades the muscularis propria (MP)	T2
T3	Tumor invades adventitia	T3
T3r	Resectable (Without adjacent structures involvement on radiograph)	–
T3br	Marginal resectable (Cannot exclude adjacent structures involvement on radiograph)	–
T4	Tumor invades adjacent structures	T4
-	Tumor invades the pleura, pericardium, azygos vein, diaphragm, or peritoneum	T4a
-	Tumor invades other adjacent structures, such as the aorta, vertebral body, or airway	T4b
-	Regional lymph nodes cannot be assessed	NX
N0	No regional lymph node metastasis	N0
N1	Metastasis in one or two regional lymph nodes	N1
N2	Metastasis in three to six regional lymph nodes	N2
N3	Metastasis in seven or more regional lymph nodes	N3
M0	No distant metastasis	M0
M1a	Metastasis in non-regional lymph nodes which can achieve acceptable outcome after dissection	M1
M1b	Metastasis in other non-regional lymph nodes and/or distant organ	M1

Following the implementation of the AJCC 8th edition, the classification of SLN metastasis as M1 has been a subject of debate. Numerous studies, leveraging database and retrospective analyses, have been conducted to challenge this categorization. Wen et al.’s SEER database analysis suggests that the impact of SLN on prognosis is contingent upon the primary tumor’s location (24). Wang’s retrospective analysis posits that SLNs should be viewed as regional nodes for upper esophageal cancer, while for middle and lower esophageal cancer, they should be considered distant metastases (25). Numata concluded through retrospective analysis that although the cervical esophagus is close to the SLN, it should still be regarded as a non-regional lymph node for patients with cervical esophageal cancer (26). Two Japanese retrospective analyses believed that the SLN does not affect the prognosis of thoracic esophageal cancer (3, 27), and should be classified as regional lymph nodes. The ongoing lack of definitive evidence sustains the controversy over the classification of SLNs, a question that future studies like Tiger study may help resolve.

**Figure 1:**
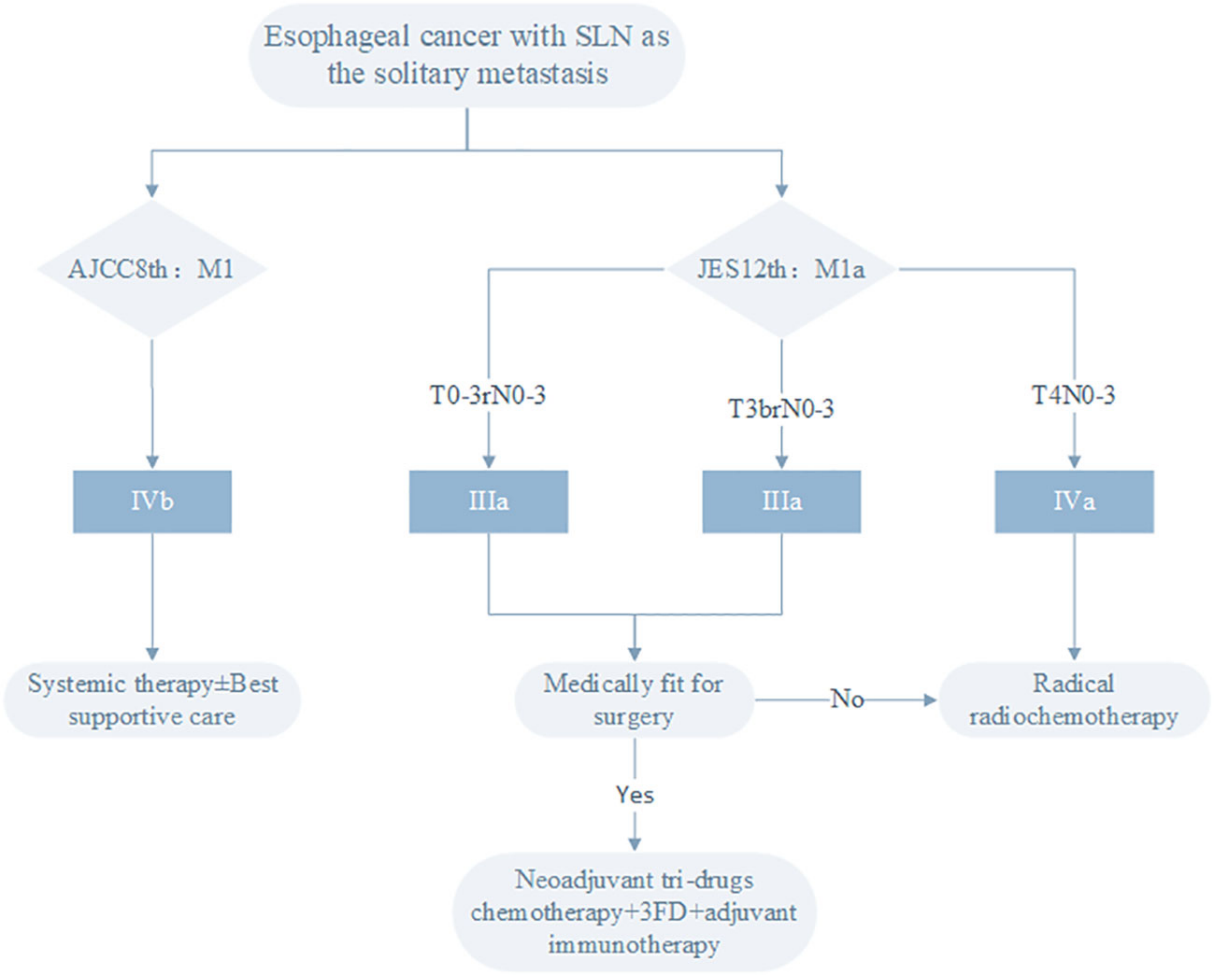
Treatment strategies for esophageal cancer patients with SLN as the solitary metastasis site based on different staging systems.

The use of different staging criteria leads to divergent treatment objectives for patients in Eastern and Western countries. The NCCN 2023 2nd Edition Esophageal Cancer Clinical Practice Guidelines recommend systemic therapy ± best supportive care for stage IVB esophageal cancer (28). China’s CSCO guidelines also use the AJCC staging, and systemic therapy is recommended for stage IVB esophageal cancer. In contrast, Japan favors a definitive treatment approach, primarily surgery or chemoradiotherapy, for patients with SLN metastasis. Based on the treatment strategies of different staging systems (**Figure 1**): In the 12th edition JES esophageal cancer staging, T0-3rN+M1a is stage IIIA, T3brAnyNM1a is stage IIIB, T4AnyNM1a is stage IVA. According to the 2022 Esophageal Cancer Diagnosis and Treatment Guidelines compiled by JES (32, 33), for stage III esophageal cancer patients who can tolerate surgery, preoperative chemotherapy or preoperative chemoradiotherapy plus surgical treatment is recommended. If the postoperative pathology does not achieve complete response, adjuvant treatment with nivolumab is recommended. Those who cannot tolerate surgery should choose radiotherapy and/or chemotherapy as the first choice. For stage IVA esophageal cancer, chemoradiotherapy is recommended, but the evidence level is not high, because there is currently no large clinical trial to prove that concurrent chemoradiotherapy (CCRT) is superior to simple chemotherapy for stage IVA patients.

### 3.2 Progress in surgical treatment

Surgical intervention remains a cornerstone in the treatment of stage III esophageal cancer. For patients with stage III according to the JES12th staging, the JES guidelines first prioritizes a regimen of tri-drug neoadjuvant chemotherapy (NCT) followed by surgery. The surgical method is esophagectomy plus three-field lymph node dissection (3FD), the 3FD in esophagectomy is defined as neck-chest-abdominal lymph node dissection, although esophageal cancer 3FD has been controversial for a long time and has not become a world standard (29, 30). For the treatment of esophageal cancer with SLN metastasis, the 3FD is an indispensable part of definitive surgery (31). Although recent years thoracoscopic or robotic surgery were able to remove left recurrent laryngeal nerve lymph nodes (32), the JES-104 group located on the lateral of the carotid vessels still requires 3FD for complete excision.

#### 3.2.1 Selection of surgical strategy

There are many studies that have confirmed that robot-assisted minimally invasive esophagectomy (RAMIE) and minimally invasive esophagectomy (MIE) are superior to open surgery (33–36), which have reduced the incidence of perioperative complications and shortened hospital stay. As for whether RAMIE is superior to MIE, based on the current research results, a preliminary affirmative answer can be obtained. The RAMIE trial is a prospective, multicenter, randomized controlled clinical trial (37), which randomly assigned patients to the RAMIE group or MIE group, the results showed: compared with the MIE group, the RAMIE group significantly shortened the operation time (203.8 mins vs 244.9 mins), for patients receiving neoadjuvant treatment, the RAMIE group had a higher efficiency of thoracic lymph node dissection (15 vs 12), and a higher completion rate of left recurrent laryngeal nerve lymph node dissection (79.5% vs 67.6%), there was no difference between the two groups in terms of bleeding volume, conversion rate and R0 resection rate, the 90-day mortality rate for both groups was 0.6%, and the overall incidence of complications was similar.

Western countries guided by the AJCC staging and NCCN guidelines are also exploring the role of surgery in stage IVb esophageal cancer with SLN as the solitary metastasis, a phase II clinical trial in the Netherlands enrolled 20 patients with cervical lymph node metastasis of esophageal cancer (38), all patients received preoperative radiotherapy plus RAMIE plus 3FD, and the 1-year overall survival(OS) rate reached 85%, a retrospective analysis based on the SEER database showed (24), the 1-year OS rate for patients with esophageal cancer with cervical lymph node metastasis undergoing non-surgical treatment was 31%, which was lower than the Netherlands study; Another retrospective study found (39), in patients with esophageal SCC with cervical lymph node metastasis, the 1-year OS rate of dCRT was 39%, which was also lower than the Netherlands study.

In summary, for esophageal cancer with SLN metastasis, the first-line treatment is 3FD based on MIE or RAMIE, and the treatment efficacy is better than non-surgery, but randomized controlled trials are still needed to further compare the pros and cons of MIE and RAMIE.

#### 3.2.2 Selection of perioperative treatment strategies

##### 3.2.2.1 Comparison of neoadjuvant tri-drugs chemotherapy and neoadjuvant chemoradiotherapy

Based on the findings of JCOG1109 (40, 41), the 2022 JES guidelines prioritize a preoperative regimen of docetaxel, cisplatin, and 5-fluorouracil (DCF) in combination with surgery over neoadjuvant chemoradiotherapy for esophageal cancer. This recommendation is based on data from patients with stages IB-III, as defined by the 7th edition of the AJCC, with the understanding that SLNs are considered regional for the upper esophageal segment, making these findings relevant to stage III patients under the current JES12 staging system (**Table 3**).

**Table 3:** Phase III study of different perioperative treatment modalities of esophageal cancer.

Author/Trial	Pathological type(%)	Clinical Stage	Treatment arms	Percentage for R0 resection(%)	pCR rate (%)	mOS	OS rate (%)	P value
OEO2(2002) (105)	SCC(31)/AC(66)	NA NA	NCT(CF)+S S	60 54	NA NA	16.8m 13.3m	23(5y) 17(5y)	0.03
JCOG9907(2012) (106)	SCC	II-III(AJCC6th)	NCT(CF)+S S+PCT	96 91	2.4 NA	NA NA	55(5y) 43(5y)	0.04
NEOCRTEC5010(2018) (53)	SCC	IIB-III(AJCC6th)	NCRT+S S	98.4 91.2	43.2 NA	100.1m 66 5m	69.1(3y) 58.9(3y)	0.025
CROSS(2021) (52)	SCC(23)/AC(75)	cT1N1M0/ cT2-3N0-1M0(AJCC6th)	NCRT+S S	92 69	29 NA	49.4m 24.0m	47(5y) /38(10y) 34(5y) /25(10y)	0.004
CheckMate577(2021) (59)	SCC(29)/ AC(71)	II-III (AJCC7th)	NCRT+S+Nivo NCRT+S	100 100	NA NA	22.4m(DFS) 10.4m(DFS)	NA NA	0.001
JCOG1109(2023) (41)	SCC	IB-III(AJCC7th)	1. NCT(CF)+S 2. NCT(DCF)+S 3. NCRT(CF)+S	84.4 85.6 87.5	2.1 19.8 38.5	4.6y NR 6.0y	62.6(3y) 72.1(3y) 68.3(3y)	1vs2 0.006 1vs3 0.12
CMISG1701(2023) (54)	SCC	cT3-4aN0-1M0(AJCC8th)	NCRT(TP)+S NCT(TP)+S	97.3 96.2	35.7 3.8	NR 43.2m	64.1(3y) 54.9(3y)	0.28
Neo-AEGIS(2023) (43)	AC	cT2-3N0-3M0	NCRT+S MAGIC/FLOT+S	95 82	16 5	NA NA	57(3y) 55(3y)	NA

NCT, neoadjuvant chemotherapy; NCRT, neoadjuvant chemoradiotherapy; NA, Not Available; CF, cisplatin + 5-fluorouracil; S, Surgery; SCC, squamous cell carcinoma; AC, adenocarcinoma; PCT, post-operative chemotherapy; TP, paclitaxel + cisplatin; NR: not reach.

Consequently, the JCOG1109 results may not be sufficient to persuade Western countries of the superiority of tri-drug neoadjuvant chemotherapy (NCT) over neoadjuvant chemoradiotherapy.

However, the JCOG1109 trial was limited to SCC patients, which contrasts with the prevalence of AC in Western populations. Previous trials, such as OEO5, have shown that four cycles of neoadjuvant epirubicin, cisplatin, and capecitabine (ECX) did not improve survival compared to two cycles of CF, and thus, do not establish a standard of care (42). Consequently, the JCOG1109 results may not be sufficient to persuade Western countries of the superiority of tri-drug NCT over neoadjuvant chemoradiotherapy.

Furthermore, the CROSS trial, which included SCC patients, did not demonstrate that neoadjuvant chemoradiotherapy was superior to perioperative FLOT chemotherapy for AC at the esophagogastric junction. The Neo-AEGIS trial (43), focusing solely on AC patients, compared the CORSS strategy with the FLOT/MAGIC strategy and found no significant difference in the 3-year OS rate, despite a higher pCR rate with the CROSS strategy. Additionally, for AC patients with HER2 overexpression, the concurrent use of trastuzumab in neoadjuvant chemoradiotherapy has not yet shown a definitive advantage (44).

##### 3.2.2.2 Patients who undergo surgery after neoadjuvant therapy still need higher level evidence to add adjuvant chemotherapy

Post-neoadjuvant therapy, esophageal cancer patients who undergo surgery require robust evidence to support the use of adjuvant chemotherapy. The survival benefits of adjuvant chemotherapy following R0 resection remain a topic of debate, with some studies suggesting no significant survival advantage (45, 46), while others indicate benefits for patients with positive postoperative lymph nodes (47), or even for those with negative lymph nodes (48, 49). The phase II PIECE trial reported a 3-year survival rate of 85% for patients receiving adjuvant S-1 therapy after NCT and R0 resection (50).

##### 3.2.2.3 Neoadjuvant radiotherapy is still the mainstream in Europe and America

The PIECE trial confirmed that R0 resection improves survival, a benefit that neoadjuvant radiotherapy can provide, translating into better outcomes. The CROSS trial (51, 52), where 75% of enrolled patients had AC and 23% had SCC. The neoadjuvant chemoradiotherapy (NCRT) group achieved a higher R0 resection rate (92% vs 69%), with a median overall survival (mOS) of 49.4 months compared to 24.0 months in the surgery group, and a 10-year survival rate of 38% and 25%, respectively. The NEOCRTEC5010 study in China (53), which included stage II-III SCC patients without supraclavicular metastasis, showed similar results, with a 3-year OS rate of 67.2%. However, the CMISG1701 trial (54), involving cT3-4aN0-1M0 ESCC patients, found no difference in the 3-year OS rate between NCT plus surgery and neoadjuvant radiotherapy plus surgery, suggesting that the CROSS strategy may not be superior to JCOG1109 in locally advanced SCC.

The evidence for the benefit of surgery in patients with SLN metastasis is limited, with neither the CROSS nor the JCOG1109 trial specifically incorporating this patient cohort. Clinical trials explicitly addressing SLN metastasis are scarce, and those that do often involve small patient numbers and are not randomized controlled trials (RCTs). A retrospective analysis in Japan revealed encouraging 5-year overall survival (OS) rates of up to 41.3% utilizing neoadjuvant radiotherapy complemented by surgery for SCC with SLN metastasis (55). In Japan’s phase II COSMOS trial (56, 57), which enrolled 48 patients with T4 or supraclavicular metastasis, the administration of DCF neoadjuvant chemotherapy (NCT) plus surgery achieved a pCR rate of 47.9% and a 1-year OS rate of 67.9%. The pCR rate and 1y-OS rate of the Dutch phase II study were 20% and 85% respectively (38), because these two phase II trials have very different pathological types and stages of research objects, they cannot be directly compared, but this suggests to us that for T4 and supraclavicular metastasis esophageal cancer patients, three-drug NCT compared to neoadjuvant radiotherapy is equally safe and effective. The JCOG1510 (58), initiated in 2019 by Japanese researchers, aims to ascertain whether three-drug NCT in conjunction with surgery or radiotherapy outperforms dCRT for esophageal cancer patients with SLN metastasis, with results pending.

In the realm of perioperative treatment strategy selection, generalizations are challenging due to regional variations in disease localization, pathological subtypes, and etiologies. In Asia and the Western world, the predominant esophageal cancer types are SCC and AC, respectively, and the clinical trial outcomes from these regions are not readily interchangeable. The absence of robust evidence leaves the optimal perioperative treatment strategy for patients with SLN metastasis undetermined, with most current treatments exploratory in nature. Future multicenter clinical trials are essential to guide more tailored and effective therapeutic approaches.

#### 3.2.3 Perioperative treatment strategy in the era of immunotherapy

Immunotherapy checkpoint inhibitors have achieved significant results in solid tumors, including esophageal cancer, sparking interest in their potential to enhance perioperative care for this disease.

While no randomized controlled trials have yet established the survival benefits of adjuvant chemotherapy following R0 resection post-neoadjuvant therapy and surgery, the CheckMate577 trial has shifted this paradigm (59). The combination of NCRT, surgery, and adjuvant immunotherapy has emerged as a novel treatment paradigm. Prior clinical trials have consistently shown that patients achieving pCR after neoadjuvant therapy and surgery exhibit significantly improved survival compared to those without pCR (60, 61). We can only analyze the near-term effects from the perspective of pCR and speculate on the long-term effects. In **Table 3**, the pCR rate of patients undergoing surgery after NCT with three drugs did not exceed 20%. Nonetheless, recent Phase II clinical trials have indicated that the integration of immunotherapy with NCT is both safe and efficacious in esophageal SCC (62–67), with pCR rates ranging from 35.8% to 50%, markedly higher than the rates achieved with two-drug NCT.

Data on the combination of NCRT, immunotherapy, and surgical treatment is currently limited to Phase II clinical trials, primarily from Asia, particularly China, and involves patients exclusively with SCC. To date, no Phase III data is available. The PERFECT study, which only enrolled AC patients, reported a pCR rate of 30% (68), The US Phase Ib clinical trial yielded a pCR rate of 40% (69); The PALACE-1 trial in China had a pCR rate of 55.6% (70), The Phase II clinical trial in South Korea had a pCR rate of 46. 1% (71). For the same enrollment of SCC, the NEOCRTEC5010 study showed that the pCR rate after NCRT was 43.2%, which was lower than the data from the PALACE-1 trial. A meta-analysis showed that NCRT combined with immunotherapy has the best pCR rate among all neoadjuvant treatment plans (72), and the pCR rate of NCRT is better than that of neoadjuvant immunotherapy combined with chemotherapy. Most of the enrollment requirements for these trials exclude patients with SLN metastasis, except for Gong’s Phase II trial (63), but the final pCR rate was not ideal, only 17.6%.

Immunotherapy has demonstrated a huge potential to improve survival in Phase I/II clinical trials and has become a first-line treatment for esophageal cancer. However, as a new treatment method, there is currently no information for patients with supraclavicular metastasis of esophageal cancer, and its improvement in survival still needs to be confirmed by the long-term follow-up results of Phase III clinical trials.

#### 3.2.4 Take home message

After the 7th edition of AJCC staging in 2009, the SLNs changed from regional lymph nodes only for cervical esophageal cancer to regional lymph nodes for all esophageal cancers. Therefore, it can be considered that clinical trials enrolled using the 7th edition of staging include patients with SLN metastasis. Based on current guidelines and clinical trial results, based on the experience in Asia, for patients with JESIII stage (SLN metastasis) esophageal SCC, neoadjuvant tri-drug chemotherapy plus three-field lymphadenectomy and adjuvant immunotherapy is currently the treatment with definite efficacy and the highest level of evidence. And based on the experience of diagnosing and treating esophageal AC in Europe and America, for AC patients, NCRT plus 3FD surgery and adjuvant immunotherapy is currently the first choice.

### 3.3 Progress in definitive chemoradiotherapy

For patients with JES12th stage III who cannot tolerate or refuse surgery, and patients with stage IVa at initial diagnosis of esophageal cancer, JES recommends dCRT (73).

The optimal management strategy for locally advanced esophageal cancer continues to be a subject of debate. Existing guidelines typically advise a course of NCRT followed by surgery, but ignore the possibility of tumor cure after chemoradiotherapy. Previous clinical trials have confirmed that nearly 40% of SCC patients have a pCR rate after NCRT. How to screen out these patients who can avoid surgery is a problem worthy of attention (**Table 4**).

**Table 4:** Clinical trials of definitive chemoradiotherapy for esophageal carcinoma.

Author/Trial	Chemotherapy	Stage	ENI/IFI	Dose	mOS	OS rate(%)	P Value
RTOG9405(2002) (75)	CF	T1-4N0-1M0	ENI	64.8Gy 50.4Gy	13m 18.1m	31(2y) 40(2y)	NA
PRODIGE5/ACCORD17 (2014) (89)	FOLFOX CF	I-IVA (AJCC6th)	ENI	50Gy	20.2m 17.5m	19.9(3y) 26.9(3y)	0.70
Chen (2019) (87)	CF TF	IIA-IVA (AJCC6th)	ENI	61.2Gy	40.3m 47.6m	40.8(5y 44.3(5y)	0.448
Lyu(2020) (82)	DP	II–III	ENI IFI	GTV60-66Gy, CTV50-50.4Gy	32.5m 34.9m	29.8(5y) 30.7(5y)	0.806
Xu(2022) (76)	DP	IIA-IVA(AJCC6th)	ENI	60Gy 50Gy	45.3m 41.2m	53.1(3y) 52.7(3y)	0.96
ESO-Shanghai 2 (2022) (88)	TF TP TC	II-IVA (AJCC6th)	ENI	61.2Gy	NA	57.2(3y) 60.1(3y) 56.5(3y)	0.77
ARTDECO(2023) (81)	TC	T1-4N0-3M0 or M1(SLN)	ENI	61.6Gy 50.4Gy	NA	39(3y) 42(3y)	0.22
You(2023) (77)	TC	II-IVb (AJCC6th)	ENI	59.4Gy 50.4Gy	28.1m 26.0m	43.5(3y) 38.1(3y)	0.54

NCT, neoadjuvant chemotherapy; NCRT, neoadjuvant chemoradiotherapy; NiCRT, neoadjuvant immune-chemoradiotherapy; NA, Not Available; CF, cisplatin + 5-fluorouracil; FOLFOX, oxaliplatin + fluorouracil + leucovorin; AJCC, American Joint Committee on Cancer; TF, paclitaxel + fluorouracil; DP, docetaxel + cisplatin; S, Surgery; SCC, squamous cell carcinoma; AC, adenocarcinoma; ENI, elective nodal irradiation; IFI, involved-field irradiation; PCT, post-operative chemotherapy; TP, paclitaxel + cisplatin; TC, paclitaxel + carboplatin; DCF: docetaxel + cisplatin + 5-fluorouracil.

Current survival data for patients with SLN metastasis treated with chemoradiotherapy are largely derived from retrospective studies with varying definitions and accuracies of SLNs. Chen et al. retrospectively analyzed 369 patients with esophageal cancer who received dCRT (74), Among them, 70 cases were combined with SLN metastasis, and 299 cases did not have SLN metastasis. The median survival of the supraclavicular metastasis group was 17.2 months, and the non-supraclavicular metastasis group was 18.4 months (p = 0.28). A similar retrospective study in the Netherlands involving 197 patients treated with chemoradiotherapy reported survival times of 23.6 months for supraclavicular metastasis and 17.1 months for non-supraclavicular metastasis (p = 0.51). These analyses suggest that SLN metastasis may not significantly impact prognosis, and that chemoradiotherapy can offer substantial survival benefits to these patients.

With the emergence of immunotherapy, research has intensified on how to optimally integrate it with chemoradiotherapy to maximize survival outcomes. Additionally, determining the optimal radiotherapy dosage, planning concurrent chemotherapy, deciding on the necessity of NCT, and evaluating the differences in adjuvant chemotherapy remain key areas of focus within the oncology community. For IVa stage patients, surgery after conversion treatment is also a hot research topic.

#### 3.3.1 Progress in radiotherapy technology

The standard radiation dose for definitive radiotherapy in esophageal cancer is currently in the range of 50-50.4Gy over 25-28 fractions, with IMRT being the preferred radiotherapy technique and Elective Nodal Irradiation (ENI) the chosen method for regional lymph node irradiation.

The relationship between escalated radiotherapy dosage and survival outcomes is a subject of ongoing debate. RTOG9405 confirmed as early as 2002 that there was no statistical difference in survival between 64.8Gy and 50.4Gy radiotherapy (75). Despite technological advancements that have reignited interest in high-dose radiotherapy, Phase III clinical trials have consistently failed to demonstrate a survival advantage, particularly for patients with SLN metastasis (76, 77).

Since increasing the dose of the target volume failed, can increasing the radiotherapy dose of the primary tumor and positive lymph nodes alone benefit patients? Can reducing the target volume still guarantee treatment efficacy? Simultaneous boost radiotherapy technology (SIB) has shown good tolerability and efficacy in some Phase I/II clinical studies. Although retrospective analysis shows that SIB tends to improve survival compared with conventional radiotherapy (78, 79), Chen’s Phase II clinical trial found that (80), locally advanced esophageal SCC, including SLN metastatic patients, received a dose of 54Gy for the subclinical lesion, and the primary tumor and lymph nodes were simultaneously boosted to 66Gy,the 5-year OS rate reached 58.4%.

However, a Phase III clinical trial published in 2021 indicated that boosting the primary lesion to 61.6Gy, as opposed to 50.4Gy, offered no additional benefit (81). It is noteworthy that this trial did not include a boost to metastatic lymph nodes. The involved-field irradiation (IFI) has been proven in Phase III clinical trials to reduce the target volume without affecting the treatment efficacy, and reduce the adverse effects of radiotherapy (82). A recent propensity score-matched study further demonstrated that for stages II-IV cervical esophageal SCC (83), IFI and ENI have equivalent therapeutic efficacy, with a reduced frequency of adverse effects in the IFI group.

The synergistic effect of SIB and IFI on survival benefits is currently under investigation in an ongoing clinical trial in China (84),which aims to provide definitive answers to these questions.

#### 3.3.2 Radiotherapy, chemotherapy, immunotherapy, how to achieve 1 + 1 + 1>3?

Researchers have been avidly exploring the optimal chemotherapy regimen for dCRT in esophageal cancer, as well as the impact of neoadjuvant and adjuvant chemotherapy. The advent of immunotherapy has further complicated and heightened the importance of these inquiries.

The results of JCOG1109 made Japanese researchers believe that DCF tri-drugs chemotherapy is a strong and effective NCT regimen for esophageal SCC (41), which is better than CF combined with radiotherapy. Therefore, some researchers retrospectively analyzed patients with advanced esophageal cancer treated with DCF regimen CCRT and found that the 5-year OS rate of patients with SLN metastasis reached 50% (85), and the 5-year OS rate of stage III patients (AJCC 8th edition) reached 76.2%. Another study published in Japan showed that the 3-year OS rate of cervical esophageal cancer treated with DCF combined with radiotherapy reached 44.2% (86), among the 18 enrolled patients, 9 were T4b and 7 were SLN metastasis. After treatment, 15 patients were CR. Two results of Phase III clinical trials reported by Chinese researchers in 2019 and 2022 respectively showed that in dCRT (87, 88), whether it is paclitaxel plus cisplatin (TP), paclitaxel plus carboplatin (TC), or paclitaxel plus 5-fluorouracil (TF), there is no survival benefit superior to the CF regimen. Therefore, TP and CF are still the first-line chemotherapy regimens for dCRT. The DCF regimen seems to have the opportunity to become a new first-line chemotherapy regimen, but it still needs to be confirmed by Phase III clinical trials. For esophageal AC, oxaliplatin plus fluorouracil plus leucovorin (FOLFOX) regimen has no survival benefit superior to the CF regimen (89).

A retrospective study that enrolled patients with SLN metastasis analyzed the differences of three different treatment strategies and found that NCT plus CCRT plus consolidation chemotherapy and NCT plus CCRT, CCRT plus consolidation chemotherapy had no survival differences (mOS 54.0m vs 35.5m vs 45.9m) (90), A meta-analysis showed that NCT or consolidation chemotherapy compared with simple CCRT can improve the 3-year OS rate (91), but the 5-year OS rate is no different. Consolidation chemotherapy can reduce the risk of distant metastasis, but these opinions need to be confirmed by clinical trials.

The efficacy of immunotherapy in dCRT for esophageal cancer is a burgeoning area of research. A phase II study in Japan (92), the TENERGY trial showed that 105 unresectable late-stage patients, including those with SLN metastasis of esophageal cancer, had a mOS of 31.0 months after dCRT followed by sequential immunotherapy with atezolizumab. A phase Ib clinical trial of carrelizumab monotherapy and definitive radiation therapy for unresectable locally advanced esophageal SCC preliminarily explored its safety and feasibility (93), with a median follow-up time of 31.0 months, the mOS and median PFS (mPFS) were 16.7 months and 11.7 months respectively. A phase II clinical trial showed that dCRT with durvalumab and tremelimumab had promising efficacy in patients with locally advanced esophageal SCC (94), the 2-year PFS rate and OS rate were 57.5% and 75% respectively. A retrospective analysis believes that the use of neoadjuvant immunotherapy plus CCRT can achieve better PFS than CCRT alone (95), the 1-year PFS rates were 72.6% and 60.0% respectively.

Regarding whether the combination of immunotherapy and radiotherapy will increase adverse effects, especially treatment-related pneumonia, the American Food and Drug Administration (FDA) has indicated that immunotherapy performed within 90 days of the patient receiving radiotherapy may not affect the occurrence of immune adverse events (96).

The integration of immunotherapy with chemoradiotherapy, whether concurrently or sequentially, and its impact on survival and adverse effects, particularly treatment-related pneumonia, is a subject of ongoing phase III clinical trials. The optimal timing and sequence of immunotherapy in relation to radiotherapy remain subjects of active investigation, with numerous studies currently in progress.

#### 3.3.3 Esophageal cancer with supraclavicular lymph node metastasis, surgery or chemoradiotherapy?

A propensity score-matching analysis from China showed that (97), for esophageal cancer with SLN metastasis, compared with the dCRT group, patients in the NCT combined with surgery group had a better 3-year OS rate (72.0% vs. 35.8%), PFS (24 vs. 14 months) and a lower 3-year tumor-related death rate (25.1% vs. 53.7%). In addition, compared with the simple radiotherapy group, patients in the dCRT group had a better 3-year survival rate (30.1% vs. 18.6%) and a lower 3-year tumor-related death rate (57.9% vs. 76.8%). A phase II randomized controlled trial compared the survival of locally advanced esophageal SCC after NCRT with clinical CR followed by dCRT or surgery (98), and there was no difference in survival between the two groups. A database-based analysis showed (99), esophageal cancer receiving dCRT plus salvage surgery and NCRT combined with surgery had no difference in survival. NEEDs trial is a multicenter phase III randomized controlled trial (100), planning to enroll 1200 patients with locally advanced esophageal SCC, comparing NCRT combined with surgery and dCRT followed by follow-up or salvage surgery, in the future can possibly answer which treatment strategy is the best for locally advanced esophageal SCC, but it is a pity that this trial did not enroll patients with SLN metastasis.

Based on the current limited clinical research conclusions, esophageal cancer patients with SLN metastasis can consider regular follow-up after achieving clinical CR with dCRT, and salvage surgery can be performed when the tumor is not controlled or recurs.

#### 3.3.4 Take home message

For esophageal cancer with SLN metastasis, if surgery is not possible or refused, dCRT is the first choice, with a radiation dose of 50-50.4Gy/25-28F, the first choice of radiotherapy technology is IMRT, for lymph nodes, IFI is recommended to reduce radiation adverse effects, concurrent chemotherapy regimens including CF or TP, are all recommended, there is no obvious benefit from adjuvant chemotherapy, adjuvant immunotherapy is widely recommended, but there is currently a lack of strong evidence to confirm its ability to improve survival. It is recommended to follow up closely after chemoradiotherapy, and timely perform salvage surgery when the tumor is not controlled or recurs.

### 3.4 Progress in systemic therapy

For esophageal cancer patients with SLN metastasis, classified as stage IVb according to the AJCC 8th edition, NCCN recommends systemic therapy with or without best supportive care. While IVb stage in JES12th (combined with other distant metastases), if the patient is combined with esophageal obstruction symptoms, still recommend chemoradiotherapy to relieve local symptoms.

For IVb stage patients with esophageal cancer combined with SLN metastasis as the only metastatic lesion, chemotherapy plus immunotherapy maybe not sufficient. A phase II clinical trial showed (101), the mOS of metastatic esophageal SCC receiving chemoradiotherapy versus simple chemotherapy was 18.3 months and 10.2 months, respectively, and the 2-year OS rate was 43.3% and 26.7%. A recent retrospective analysis showed (102), the 5-year OS of metastatic esophageal SCC receiving chemoradiotherapy was better than simple chemotherapy, 17.6% and 8.2% respectively. Thus, whether it is SLNs or combined with other distant metastases, chemoradiotherapy is better than chemotherapy.

#### 3.4.1 Optimal first-line chemotherapy regimens

NCCN adopts different chemotherapy regimens for different pathological types (28). JES guidelines just recommended chemotherapy regimen SCC, which is main pathological type in the East Asian population (73), and no regimen have been developed for other pathological types. JES recommends CF plus PD-1 inhibitor for all stage IVb esophageal cancers, while NCCN guidelines recommend the use of oxaliplatin instead of cisplatin to reduce chemotherapy related adverse events, and the first-line recommended chemotherapy regimen for SCC patients is 5-fluorouracil plus oxaliplatin plus PD-1 inhibitor, for AC, depending on whether HER-2 is overexpressed to decide whether to add trastuzumab or not.

Currently, there is a dearth of clinical trials or retrospective analyses specifically addressing chemotherapy for esophageal cancer patients with SLN metastasis.

According to existing guidelines, the first-line regimen for esophageal cancer chemotherapy alone involves a combination of 5-fluorouracil, cisplatin or oxaliplatin, and a PD-1 inhibitor. For AC patients with HER2 overexpression, trastuzumab may be incorporated. For patients with PD-L1 expression of 1% or more, a dual immunotherapy regimen of nivolumab plus ipilimumab may be considered.

#### 3.4.2 Progress in targeted therapy

The 2022 guidelines for the diagnosis and treatment of esophageal cancer compiled by JES believe (73), in addition to immunotherapy, there is currently not enough evidence to show that first-line chemotherapy combined with cetuximab, panitumumab and other EGFR monoclonal antibodies can improve survival, EGFR-TKI, such as gefitinib compared with placebo also did not achieve survival benefit, the guidelines did not mention other targeted drugs. The latest version of the NCCN guidelines mentions several targets such as Her-2, VEGFR, NTRK, PD-1, CTLA-4, RET, BRAF V600E (28), the corresponding targeted drugs have all demonstrated effectiveness in clinical trials, but currently except for Her-2 targeted drug trastuzumab and immune checkpoint inhibitors:pembrolizumab, nivolumab, ipilimumab, dostarlimab-gxly can be used as first-line drugs, the rest are all second-line treatment drugs.

Zolbetuximab, a novel monoclonal antibody, targets the Claudin-18 isoform 2 (CLDN18.2) protein, which is expressed in both normal gastric cells and malignant gastric or EGJ adenocarcinoma cells. Zolbetuximab has been proven to be effective in CLDN18.2 positive EGJ adenocarcinoma (103), combining zolbetuximab with modified folinic acid, fluorouracil, and oxaliplatin regimen (mFOLFOX6), compared to using mFOLFOX6 alone, the combination treatment group had a mPFS of 10.61 months, compared to 8.67 months in the control group, which may become a new first-line treatment option for these patients. Another study targeted Her-2 negative locally advanced unresectable or metastatic gastric or EGJ adenocarcinoma patients (104), zolbetuximab combined with capecitabine and oxaliplatin as first-line treatment, significantly improved PFS and OS, 8.21 months and 14.39 months compared to 6.80 months and 12.16 months. Therefore, this treatment combination may become a new first-line treatment option for Her-2 negative advanced gastric or EGJ adenocarcinoma patients.

For esophageal SCC, EGFR monoclonal antibodies and TKIs can be considered for second-line and subsequent treatment. For esophageal AC, there are currently several new targeted drugs proven to be effective, but as first-line treatment still needs further confirmation from clinical trials, at present, immune checkpoint inhibitors and Her-2 targeted drugs are still the main force in targeted therapy for esophageal cancer.

#### 3.4.3 Take home message

Esophageal cancer with SLN metastasis, in the AJCC8th staging, is stage IVb. It is recommended to perform second-generation sequencing to detect gene mutations, and choose different first-line treatment regimen based on pathological types and gene detection results. The first-line regimen for SCC patients is CF combined with PD-1 inhibitor. For AC patients, the regimen consists of 5-fluorouracil, oxaliplatin, and a PD-1 inhibitor, with the addition of trastuzumab for HER2-overexpressing tumors.

## 4 Summary and future perspectives

The treatment of esophageal cancer, particularly for patients with SLN metastasis, continues to present significant challenges. The variability in pathological types and the lack of consensus on SLN definitions and staging systems between East Asia and Western countries have led to diverse treatment strategies. The forthcoming results of the Tiger study are anticipated to provide clarity and standardization in this regard (26).

In East Asia, where ESCC is prevalent, the approach to treating patients with SLN metastasis draws from experiences that emphasize comprehensive treatment strategies. These may include radiotherapy or surgery-based treatments, with decisions informed by the staging outlined in the 12th edition of the JES guidelines. Conversely, in Western countries, where AC is more common, the 8th edition of the AJCC classifies SLN metastasis as distant metastasis, advocating for systemic therapy as the initial approach.

Emerging evidence supports the notion that a multidisciplinary approach, incorporating either radiotherapy or surgery, can yield superior outcomes compared to chemotherapy alone. As we look to the future, the integration of immune checkpoint inhibitors with existing treatment modalities is a promising avenue that warrants further exploration. The optimal sequencing and combination of these agents with other therapies remain critical issues to address, as the widespread adoption of immunotherapy has highlighted the need for strategies that maximize clinical benefit while minimizing adverse effects.

Targeted therapy for ESCC is another burgeoning research area. While the combination of chemoradiation and targeted therapy has shown potential to enhance treatment efficacy and reduce drug resistance, the current incidence of adverse events associated with such combination treatments is still concerning. The complex interplay among signaling pathways targeted by these therapies suggests a need for caution to prevent unforeseen complications. Therefore, the development of novel targeted therapies must prioritize the assessment of adverse events as a key factor in ensuring patient safety and treatment tolerability.

In summary, as we await the definitive findings of the Tiger study to guide more uniform and effective treatment strategies, the ongoing evolution of therapeutic options for esophageal cancer, including the integration of immunotherapy and targeted agents, holds great promise for the future management of this disease.
